# Dysbiosis of Intestinal Microbiota and Decreased Antimicrobial Peptide Level in Paneth Cells during Hypertriglyceridemia-Related Acute Necrotizing Pancreatitis in Rats

**DOI:** 10.3389/fmicb.2017.00776

**Published:** 2017-05-04

**Authors:** Chunlan Huang, Jing Chen, Jingjing Wang, Hui Zhou, Yingying Lu, Lihong Lou, Junyuan Zheng, Ling Tian, Xingpeng Wang, Zhongwei Cao, Yue Zeng

**Affiliations:** ^1^Department of Gastroenterology, Shanghai General Hospital, Shanghai Jiao Tong University School of MedicineShanghai, China; ^2^Shanghai Key Laboratory of Pancreatic Diseases, Shanghai General Hospital, Shanghai Jiao Tong University School of MedicineShanghai, China; ^3^International Medical Care Center, Shanghai General Hospital, Shanghai Jiao Tong University School of MedicineShanghai, China

**Keywords:** hypertriglyceridemia, acute necrotizing pancreatitis, Paneth cell, antimicrobial peptides, intestinal microbiota

## Abstract

Hypertriglyceridemia (HTG) aggravates the course of acute pancreatitis (AP). Intestinal barrier dysfunction is implicated in the pathogenesis of AP during which dysbiosis of intestinal microbiota contributes to the dysfunction in intestinal barrier. However, few studies focus on the changes in intestine during HTG-related acute necrotizing pancreatitis (ANP). Here, we investigated the changes in intestinal microbiota and Paneth cell antimicrobial peptides (AMPs) in HTG-related ANP (HANP) in rats. Rats fed a high-fat diet to induce HTG and ANP was induced by retrograde injection of 3.5% sodium taurocholate into biliopancreatic duct. Rats were sacrificed at 24 and 48 h, respectively. Pancreatic and ileal injuries were evaluated by histological scores. Intestinal barrier function was assessed by plasma diamine oxidase activity and D-lactate level. Systemic and intestinal inflammation was evaluated by tumor necrosis factor alpha (TNFα), interleukin (IL)-1β, and IL-17A expression. 16S rRNA high throughput sequencing was used to investigate changes in intestinal microbiota diversity and structure. AMPs (α-defensin5 and lysozyme) expression was measured by real-time polymerase chain reaction (PCR) and immunofluorescence. The results showed that compared with those of normal-lipid ANP (NANP) groups, the HANP groups had more severe histopathological injuries in pancreas and distal ileum, aggravated intestinal barrier dysfunction and increased TNFα, IL-1β, and IL-17A expression in plasma and distal ileum. Principal component analysis showed structural segregation between the HANP and NANP group. α-Diversity estimators in the HANP group revealed decreased microbiota diversity compared with that in NANP group. Taxonomic analysis showed dysbiosis of intestinal microbiota structure. In the HANP group, at phyla level, *Candidatus_Saccharibacteria* and Tenericutes decreased significantly, whereas Actinobacteria increased. At genus level, *Allobaculum*, *Bifidobacterium*, and *Parasutterella* increased significantly, while *Alloprevotella*, *Anaerotruncus*, *Candidatus_Saccharimonas*, *Christensenellaceae_R-7_group*, *Rikenellaceae_RC9_gut_group*, *Ruminiclostridium_5*, *Ruminococcaceae_UCG-005*, and *Ruminococcaceae_UCG-014* decreased. Compared with those in the NANP rats, mRNA expression of lysozyme and α-defensin5 and protein expression of lysozyme decreased significantly in the HANP rats. Moreover, in the NANP rats and the HANP rats, *Allobaculum* abundance was inversely correlated with lysozyme expression, while *Anaerotruncus* abundance was positively correlated with it by Spearman test. In conclusion, intestinal microbiota dysbiosis and decreased AMPs of Paneth cells might participate in the pathogenesis of intestinal barrier dysfunction in HANP.

## Introduction

Hypertriglyceridemia (HTG) is a well-established risk factor for acute pancreatitis (AP). It is the third most common cause of AP and accounts for up to about 10% of all cases of pancreatitis episodes (Valdivielso et al., 2014). Clinical studies reported that acute necrotizing pancreatitis (ANP) patients with HTG suffered a more severe clinical course and complications including infection, sepsis, and multiple organ failure. The animal experiments also gave the evidences that HTG with AP could exaggerate pancreatic and systemic inflammatory response (Ponzo et al., 2006; Zeng et al., 2012; Zheng et al., 2016). Nevertheless, it has not been fully elucidated how the HTG influences and deteriorates the course of AP.

The disorder of intestinal microbiota has been proved to be relative to the intestinal barrier dysfunction in many diseases, such as inflammatory bowel disease (Sekirov et al., 2010), alcoholic live disease (Kirpich et al., 2016), and kidney disease (Sabatino et al., 2015). In AP, intestinal microbiota dysbiosis is also one of the factors that influence the intestinal barrier (Zhang et al., 2007). Moreover, it has been reported that patients with severe AP (SAP) had dysbiosis of intestinal microbiota, including reduced microbiota diversity, increased *Enterococcus* and Enterobacteriaceae, and decreased *Bifidobacterium* (Tan et al., 2015). High-fat diet (HFD) is a factor that influences the composition of intestinal microbiota, and leads to low-grade intestinal inflammation and increased intestinal permeability (de La Serre et al., 2010). However, few studies focus on the alteration of intestinal microbiota in HTG-related ANP (HANP).

The intestinal barrier comprises several functional and structural components, including the intestinal microbiota, digestive enzymes, unstirred water and mucous layer and epithelial layer (Flint and Windsor, 2003). The intestinal epithelial layer consists of four main specialized cell lineages: absorptive enterocytes and three secretory cell types: enteroendocrine, Paneth, and goblet cells. Among these cell types, Paneth cells play a pivotal role in the maintenance of intestinal barrier function (Clevers and Bevins, 2013). These cells produce and secret various antimicrobial peptides (AMPs) including lysozyme and defensins, and contribute to shape the composition of intestinal microbiota in intestine (Nakamura et al., 2016). Study has indicated that deficiency in Paneth cell AMPs was associated with intestinal barrier failure, resulting in the bacterial translocation (Teltschik et al., 2012). Impaired Paneth cells also led to inflammation in gut (Adolph et al., 2013). Reduced production of α-defensin in Paneth cells were found in patients with Crohn’s disease and it was considered that the decrease in α-defensin might compromise antimicrobial defenses of intestinal mucosa and lead to changes in structure of intestinal bacteria which predisposed to the inflammation of ileal Crohn’s disease (Yvon et al., 2016). In HANP, however, there are few studies on Paneth cells.

In this study, we investigated and analyzed the inflammation and injuries in small intestine, the alterations of intestinal microbial structure, and the expression of Paneth cell AMPs during HANP. The results suggested that the intestinal barrier dysfunction in HANP was accompanied with the disorder in intestinal microbiota and the decrease in Paneth cell AMPs, indicating the crucial role of intestinal microbiota and Paneth cells in pathogenesis of HANP through disturbing the intestinal barrier.

## Materials and Methods

### Animals

All the animal experimental protocols were approved by the Animal Care and Use Committee of Shanghai Jiao Tong University and the experiments were performed in accordance with the guidelines of the committee (approving number: 20130050).

Male Sprague-Dawley rats weighing 120–150 g were purchased from Shanghai Laboratory Animal Co. Ltd (SLAC; Shanghai, China). All rats were housed at a constant room temperature 24°C with a 12-h light–dark cycle and had free access to water and rat chow. Every five rats were housed in a cage.

**FIGURE 1:**
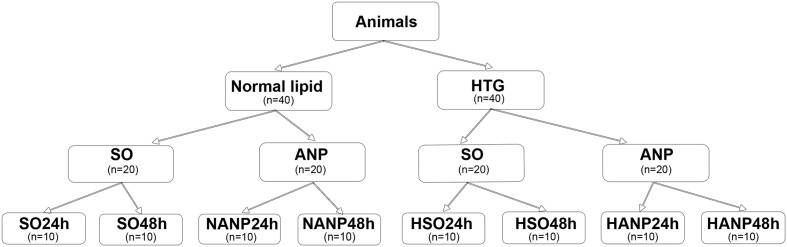
**Study design.** SO, sham-operated; ANP, acute necrotizing pancreatitis; SO24h, normal lipid + sham-operated + sacrificed at 24 h; SO48h, normal lipid + sham-operated + sacrificed at 48 h; NANP24h, normal lipid + ANP + sacrificed at 24 h; NANP48h, normal lipid + ANP + sacrificed at 48 h; HSO24h, HTG + sham-operated + sacrificed at 24 h; HSO48h, HTG + sham-operated + sacrificed at 48 h; HANP24h, HTG + ANP + sacrificed at 24 h; HANP48h, HTG + ANP + sacrificed at 48 h.

The experimental design is shown in **Figure 1**. Eighty Sprague-Dawley rats were randomly allocated to two groups (normal lipid and HTG, *n* = 40, respectively). Rats with normal lipid were divided into sham-operated (SO) group and ANP group with 20 rats in each group. Each group was then divided into 24h and 48h subgroups, respectively (SO24h, SO48h, NANP24h, and NANP48h, each group *n* = 10). Rats with HTG were divided into SO group and ANP group with 20 rats in each group. Each group was then divided into 24h and 48h subgroups, respectively (HSO24h, HSO48h, HANP24h, and HANP48h, each group *n* = 10).

HTG rats were fed with HFD (77% normal chow + 20% saturated animal fat, lard + 3% cholesterol) and the normal lipid rats with normal chow. After 2 weeks of HFD, ANP was induced by retrograde injection of 3.5% sodium taurocholate solution at a volume of 1 ml/kg into the biliopancreatic duct. Rats with sham-operation were injected with saline instead. Rats were sacrificed at 24 and 48 h after ANP induction. Blood samples were collected from abdominal aorta. The pancreas and the distal ileum were promptly fixed in 10% neutral buffered formaldehyde solution for further histological examination. Segments of the distal ileum were isolated, washed with cold phosphate-buffered saline (PBS), snap-frozen in liquid nitrogen, and stored at -80°C for further experiments. Fresh feces were collected from transected 5–6 cm portion of the distal ileum. The ileum was opened longitudinally. The fecal materials were collected, immediately snap-frozen in liquid nitrogen, and stored at -80°C until further analysis.

### Serum Biochemistry

Serum levels of triglyceride (TG) and total cholesterol (TC) were analyzed using the automated biochemical analyzer (Advia 1650, Bayer, Germany).

### Histological Examination

For light microscope observation, the pancreatic and ileal tissues fixed in 10% neutral buffered formaldehyde solution were dehydrated, embedded in paraffin and then cut into 4 μm sections. The sections were routinely stained with hematoxylin and eosin, and observed by two pathologists who were blinded to the experiment via a light microscope. The morphologic changes of the pancreas, including pancreatic edema, acinar cell necrosis, adipose necrosis, hemorrhage, and inflammation, were scored according to the criteria of Schmidt (Schmidt et al., 1992). The pathological damage of the ileum was graded according to the Chiu’s standard (Chiu et al., 1970).

### Analysis of Plasma D-Lactate and Diamine Oxidase

To evaluate the severity of intestinal barrier dysfunction, plasma diamine oxidase (DAO) and D-lactate were measured as indicators of intestinal mucosal mass and integrity. DAO activity was measured with a commercial kit (Nanjing Jiancheng Bioengineering Institute, China) according to the manufacturer’s instruction. Plasma D-lactate level was determined by using a commercial kit (Abcam, UK) in accordance with the manufacturer’s protocol.

### Analysis of TNFα, IL-1β, and IL-17A Levels in the Plasma and the Distal Ileum

The expression levels of inflammatory cytokines tumor necrosis factor alpha (TNFα), interleukin (IL)-1β, and IL-17A in circulation and small intestine were measured by analyzing plasma and distal ileum tissue. One centimeter long segment of the distal ileum was dissected, washed, snap-frozen in liquid nitrogen, and stored at -80°C. Intestinal tissue with equal weight (100 mg) from each group was homogenized with 1 ml PBS containing 1% protease inhibitor. The homogenates were centrifuged at 12,000 rpm at 4°C for 5 min. The supernatants were collected in sterile tubes and stored in -80°C. The concentration of total protein was detected using bicinchoninic acid (BCA) protein assay kit (Beyotime, China). TNFα, IL-1β, and IL-17A levels in plasma and distal ileum tissue was measured by enzyme-linked immunosorbent assay (ELISA) (eBioscience, USA).

### DNA Extraction, PCR Amplification, Sequencing, and Bioinformatics Analysis

Bacterial DNA was extracted from 200 mg rat feces per sample using the E.Z.N.A. Soil DNA Kit (Omega Bio-Tek, Norcross, GA, USA) according to the manufacturer’s protocols. The concentration of DNA was measured using a NanoDrop2000 (Thermo Scientific, Waltham, MA, USA). Bacterial DNA was amplified with 338F (ACTCCACGGGAGGCA) and 806R (GGACTACHVGGGTWTCT) primers covering V3–V4 region of the bacterial 16S rRNA gene. Polymerase chain reaction (PCR) amplification was conducted in a 20 μl reaction volume, including 10 ng of DNA template, 4 μl of FastPfu Buffer, 2 μl of dNTPs (2.5 mM), 0.8 μl of each primer (5 μM) (Sangon Biotech, Shanghai, China) and 0.4 μl of FastPfu Polymerase (TransStart^®^ FastPfu DNA Polymerase, TransGen BioTech, Beijing, AP221-01). The PCR products were checked with 2% (wt/vol) agarose gel electrophoresis and purified with the AxyPrep DNA Gel Extraction Kit (Axygen Biosciences, Union City, CA, USA) according to the manufacturer’s introductions and quantified via QuantiFluor-ST (Promega, Madison, WI, USA). Equimolar concentrations of amplifications were pooled and sequenced on an Illumina MiSeq platform.

The raw sequencing reads were denoised and quality-filtered using Trimmomatic and FLASH software. The high-quality sequences were assigned to samples according to barcodes. Operational taxonomic units (OTUs) were clustered at 97% nucleotide similarity level using UPARSE (version 7.1)^1^ and chimeric sequences were identified and removed using UCHIME. The taxonomy of each 16S rRNA gene sequence was analyzed by RDP Classifier^2^ against the SILVA 119 16S rRNA database using a confidence threshold of 70%. OTUs that reached 97% similarity were used for alpha diversity estimations, including diversity (Shannon, Simpson), richness (Chao 1), and Good’s coverage and rarefaction curve analysis using Mothur (Version 1.30.2)^3^. The principal component analysis (PCA) was generated in accordance with the Euclidean calculated using OTU information from each sample and the heatmap was generated with gplot package of R software.

### Expression of Paneth Cell AMPs by Real-Time PCR and Immunofluorescence

#### RNA Isolation and Real-Time PCR

Total RNA was isolated from segments of the distal ileum using TRIzol kit (Invitrogen, USA). Reverse transcription (RT) was conducted using PrimeScript RT master mix kit (Perfect Real Time kit, TaKaRa, Shige, Japan). The real-time PCR analysis of lysozyme and α-defensin5 expression was performed via SYBR Premix Ex Taq^TM^ kit (TaKaRa, Shiga, Japan) following the manufacturer’s instructions. Relative values of gene expression were analyzed using 2^-ΔΔCt^ method. All primers were synthesized by Sangon (Shanghai, China). The sequences of primers were as follows: forward 5′-AGGAATGGGATGTCTGGCTAC-3′ and reverse 5′-GGTATCCCACAGGCGTTCTT-3′ for lysozyme, forward 5′-TCCAGCGCATGAAGACACTT-3′ and reverse 5′-GTCTCAGCGGCAACAGAGTA-3′ for α-defensin5. β-actin was used as an internal control: forward 5′-AGGATGCAGAAGGAGATTACTGC-3′ and reverse 5′-AAACGCAGCTCAGTAACAGTGC-3′. All experiments were performed in triplicate.

#### Immunofluorescence

Immunofluorescence analysis was performed to measure the protein expression level of lysozyme in Paneth cells. Paraffin-embedded sections of the distal ileum were deparaffinized and rehydrated, and then antigen retrieval was performed in citrate buffer. Endogenous peroxidase activity was blocked using 3% hydrogen peroxide in methanol for 15 min, and then the slides were incubated with primary antibody at 4°C overnight. The antibody used for single staining was as follows: anti-lysozyme (1:1000 dilution, Dako, Denmark). After washing, the sections were incubated with fluorescein-labeled secondary antibody for 30 min. DNA was stained with dihydrochloride (DAPI) for 5 min to visualize nucleus.

#### Data Availability

The 16S rDNA sequence data generated in this study were submitted to the GenBank Sequence Read Archive accession number SRP081324.

### Statistical Analysis

Data are presented as mean ± standard deviation (SD). Student’s *t*-test and Mann–Whitney test and Spearman test were performed using SPSS 19.0 software and a *p*-value of <0.05 was considered statistically significant.

## Results

### HTG Aggravated Pathological Changes in the Pancreas and the Distal Ileum in ANP

**FIGURE 2:**
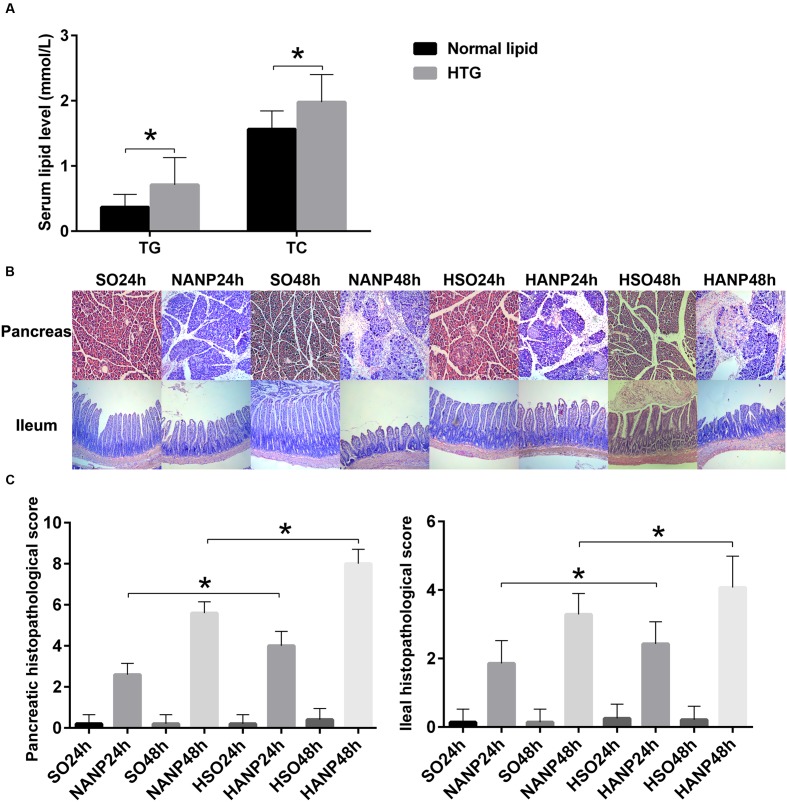
**HTG aggravated pathological changes in the pancreas and the distal ileum in ANP.** **(A)** Serum TG and TC levels in normal lipid and HTG groups. **(B)** Histopathological changes in the pancreas and the distal ileum of rats [hematoxylin-eosin (HE), ×100]. **(C)** Histopathological scores of the pancreas and the distal ileum. ^∗^*p* < 0.05. Student’s *t*-test.

Rats were fed with a HFD for 2 weeks to establish a hyperlipidemia model. Compared with those of the normal diet group, serum levels of TG and TC significantly increased by 130 and 27% in the HFD rats, respectively (*p* < 0.05; **Figure 2A**).

In all ANP rats, the pancreatic injuries were featured by extensive enlarged interlobular interspaces, patchy necrosis, hemorrhage, and inflammatory cell infiltration. The injuries in pancreas had higher histopathological scores in the HANP groups than those in the normal-lipid ANP (NANP) groups (*p* < 0.05). We also found the histopathological changes in distal ileum including shortened villi, edema, and infiltration of inflammatory cells in rats with ANP. In consistence with the histopathology of pancreas, the distal ileum in the HANP groups also had higher pathological scores compared with those of the NANP groups (*p* < 0.05). Both pancreas and ileum had more severe damage and higher histopathological scores in the HANP48h group (**Figures 2B,C**).

### HTG Aggravated Changes in Intestinal Barrier Permeability and the Expression of Inflammation Cytokines in the Plasma and the Distal Ileum in ANP

**FIGURE 3:**
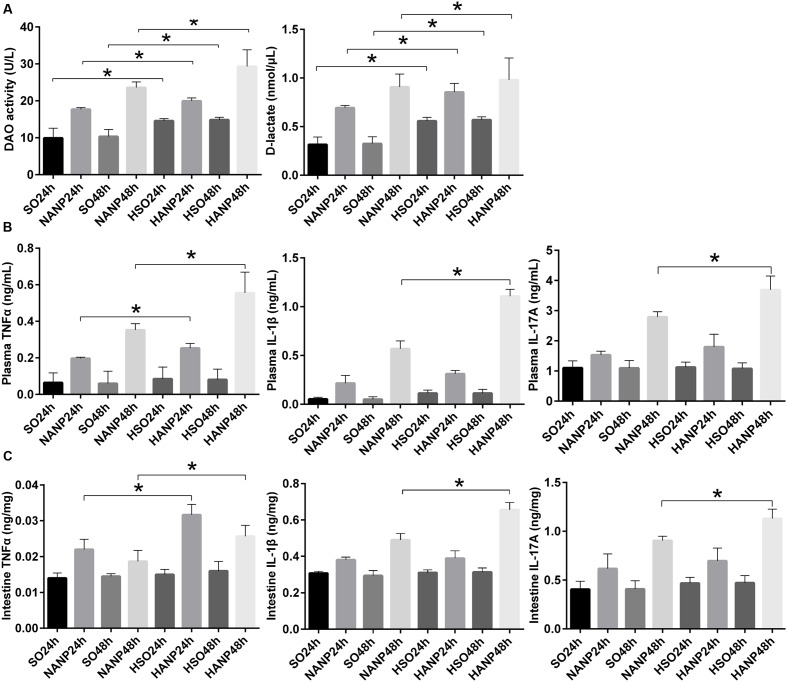
**HTG aggravated changes in intestinal barrier permeability and the expression of inflammation cytokines in plasma and the distal ileum in ANP.** **(A)** Measurement of plasma diamine oxidase (DAO) activity and D-lactate at 24 and 48 h after ANP induction. **(B)** Plasma inflammation cytokines TNFα, IL-1β, and IL-17A expression levels in rats 24 and 48 h after ANP induction. **(C)** Intestinal inflammatory cytokines TNFα, IL-1β, and IL-17A expression levels at 24 and 48 h after ANP induction. ^∗^*p* < 0.05. Student’s *t*-test.

Plasma DAO and D-lactate were measured to evaluate the severity of intestinal barrier dysfunction. In consistence with previous study, intestinal barrier had injury after ANP induction characterized by significantly increased DAO and D-lactate expression levels (Liang et al., 2014). Compared with those in the SO groups, DAO and D-lactate expression levels significantly increased in the HSO groups. The expression levels of DAO and D-lactate in the HANP groups at 24h and 48h were higher than those in the NANP groups at the same time points (*p* < 0.05). The HANP 48h group had the highest expression levels of plasma DAO and D-lactate among these groups (**Figure 3A**).

TNFα plays a key role in SAP, triggering expression of other inflammatory cytokines such as IL-1β and aggravating the tissue injury. IL-1β is required for the production of IL-17 (Shaw et al., 2012). They are all important proinflammatory cytokines in AP. We measured TNFα, IL-1β, and IL-17A expression levels in system and intestine by ELISA. As shown in **Figure 3B**, plasma inflammatory cytokines increased after ANP induction. Compared to those in the SO groups, plasma TNFα, IL-1β, and IL-17A expression levels elevated in the HSO groups, but no statistical significance was attained. Compared with that in the NANP groups, plasma TNFα level increased significantly in the HANP groups (*p* < 0.05, respectively). Plasma IL-1β and IL-17A levels in the HANP group at 48h significantly increased compared with those in the NANP at 48h and were higher than those in the HANP24h group (*p* < 0.05). We also evaluated the intestinal inflammatory cytokines expression. As previous study demonstrated (Yin et al., 2011), intestinal inflammatory cytokines increased after ANP induction. Compared to those in the SO groups, intestinal TNFα, IL-1β, and IL-17A expression levels in the HSO groups elevated, but there was no statistical significance. Compared with that in the NANP groups, intestinal TNFα level increased significantly in the HANP groups (*p* < 0.05, respectively). Intestinal IL-1β and IL-17A levels significantly increased in the HANP group at 48h compared to those in the NANP48h group and were higher than those in the HANP24h group (*p* < 0.05; **Figure 3C**). These findings indicated that the intestine of HANP rats had more severe inflammation.

### Changes in Intestinal Microbiota Diversity and Structure in HTG-Related ANP

**FIGURE 4:**
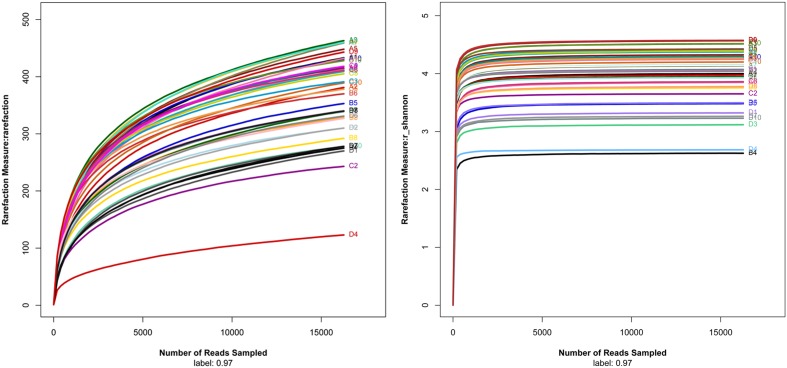
**Rarefaction analysis and Shannon diversity of all samples**.

The intestinal histopathological changes and intestinal barrier injuries were more severe at 48h after ANP induction. Therefore, we analyzed the intestinal microbiota in the SO48h, NANP48h, HSO48h, and HANP48h groups. A total of 650,600 high-quality sequences and 910 OTUs were obtained from the forty samples using sequencing. The rarefaction curves tended to approach the saturation plateau and the Shannon diversity of all samples was stable, suggesting that most diversity had already been discovered (**Figure 4**).

**FIGURE 5:**
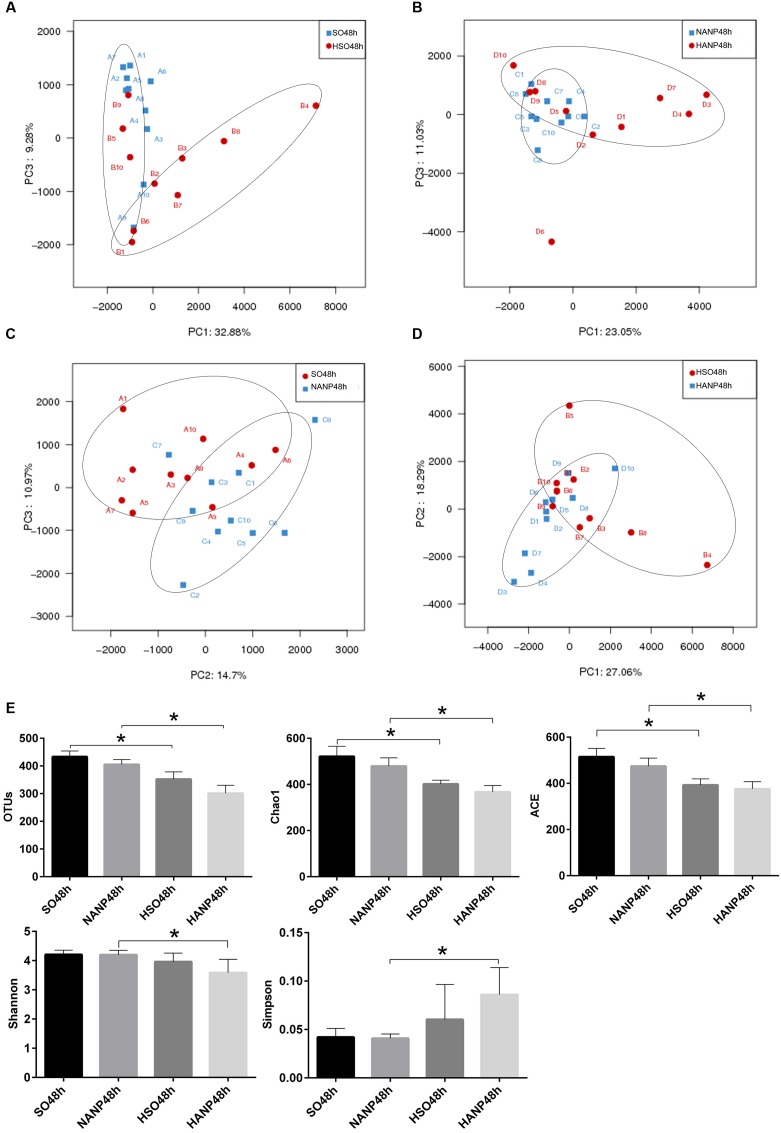
**Changes in intestinal microbiota diversity in HTG-related ANP.** **(A–D)** β-diversity reflected by PCA among the four groups. **(E)** The estimators of α-diversity of intestinal microbiota in each group. ^∗^*p* < 0.05. Student’s *t*-test.

PCA based on abundance of OTU was performed to provide an overview of the intestinal microbiota composition. As shown in **Figure 5A**, PC1 and PC3, accounting for 32.88 and 9.28%, respectively of total variance, reflected a separation of the SO48h and HSO48h groups. In **Figure 5B**, PC1 and PC3, accounting for 23.05 and 11.03%, respectively of total variance, showed separated microbiota composition between the NANP48h and HANP48h groups. The **Figure 5C** revealed separated microbiota composition of the SO48h and NANP48h groups based on PC2 and PC3 which accounted for 14.7 and 10.97%, respectively of total variance. In **Figure 5D**, PC1 and PC2 (27.06 and 18.29% of total variance, respectively) reflected different microbiota community between the HSO48h and HANP48h groups. Principal coordinate analysis (PCoA) and nonmetric multidimensional scaling (NMDS) reflected the same results (Supplementary Data).

The estimators of community α-diversity, shown in **Figure 5E**, reflected that intestinal microbiota diversity decreased in the HSO48h group and the HANP48h group had the lowest diversity among the four groups. Compared to that in the SO48h group, OTU, Chao 1 and ACE index reflected significantly decreased intestinal microbiota diversity in the HSO48h group (*p* < 0.05). The intestinal microbiota diversity in the HANP48h group significantly decreased compared to that in the NANP48h group, which was demonstrated by the estimators of OTU, Chao 1, ACE, Shannon, and Simpson index.

**FIGURE 6:**
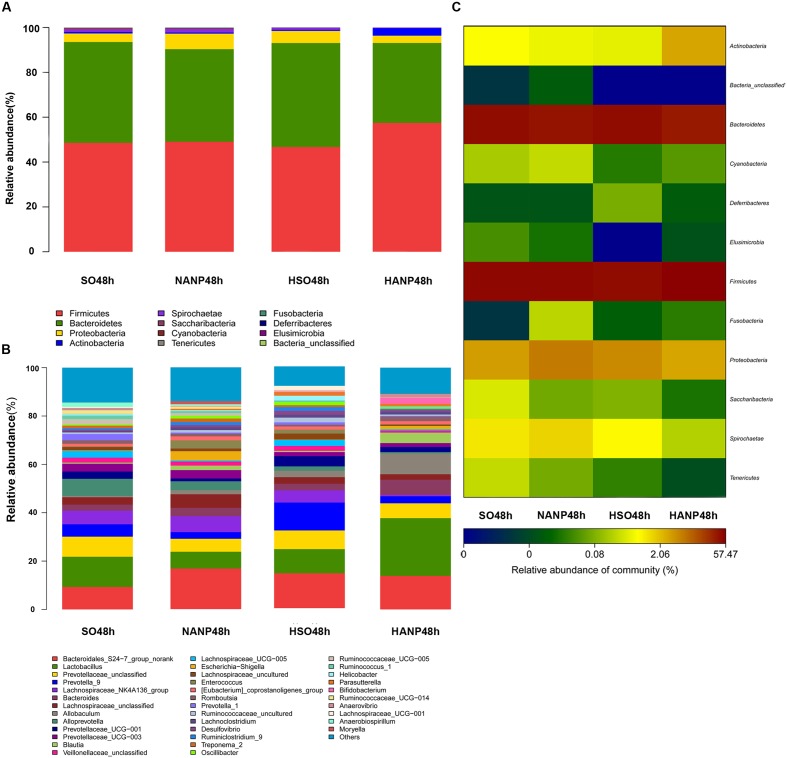
**(A)** Relative abundance of phyla in intestinal microbiota of the four groups. **(B)** Relative abundance of genera in intestinal microbiota of the four groups. **(C)** The heatmap of bacterial phyla among the four groups.

**FIGURE 7:**
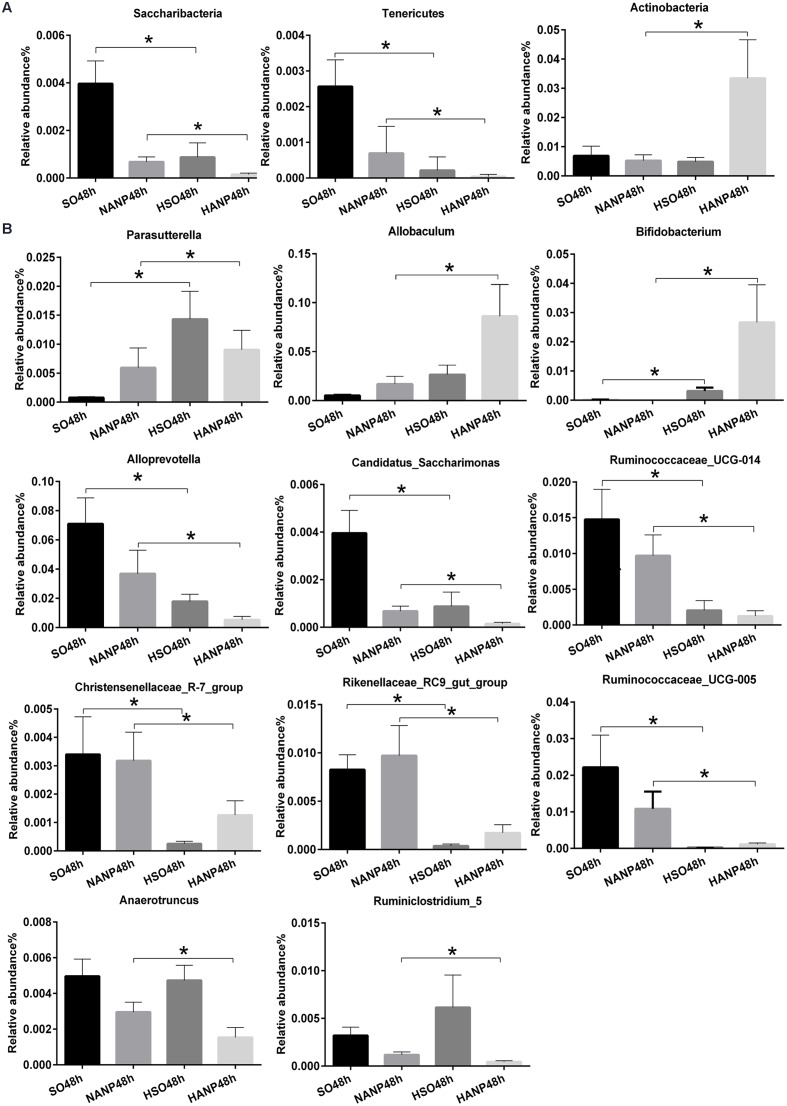
**Relative abundance of significantly different phyla and genera among the four groups.** **(A)** The significantly different phyla among the four groups. **(B)** The significantly different genera among the four groups. ^∗^*p* < 0.05. Mann–Whitney test.

**Figure 6** reflected that there was an overall shift in intestinal microbiota structure. In the phyla level, the HSO48h group had significant lower abundance of *Candidatus_Saccharibacteria* and Tenericutes compared with that of the SO48h group (*p* < 0.05). After induction of ANP, the abundance of these two phyla further reduced. Their abundance was the lowest in the HANP48h group and significantly decreased compared with that in the NANP48h group (*p* < 0.05). The HANP48h group had the highest abundance of Actinobacteria among the four groups and significantly increased compared with that in the NANP48h group (*p* < 0.05; **Figure 7A**). In the genus level, the abundance of *Parasutterella*, *Allobaculum*, and *Bifidobacterium* in the HSO48h group was higher than that in the SO48h group, whereas only the increases in *Parasutterella* and *Bifidobacterium* were statistically significant (*p* < 0.05). After ANP induction, their abundances significantly increased in the HANP48h group compared with that in the NANP48h group (*p* < 0.05). The abundance of *Alloprevotella*, *Candidatus_Saccharimonas*, and *Ruminococcaceae_UCG-014* significantly decreased in the HSO48h group compared with that of the SO48h group (*p* < 0.05). After ANP induction, the abundance of all these three genera decreased in the HANP48h group, which was the lowest among the four groups. Compared with the SO48h group, the abundance of *Christensenellaceae_R-7_group*, *Rikenellaceae_RC9_gut_group*, and *Ruminococcaceae_UCG-005* significantly decreased in the HSO48h group (*p* < 0.05). Their abundance altered after ANP induction and was significantly decreased in the HANP48h group compared with that in the NANP48h group (*p* < 0.05). The abundance of *Anaerotruncus* and *Ruminiclostridium_5* decreased after ANP surgery and significantly decreased in the HANP48h group compared with that in the NANP48h group (*p* < 0.05; **Figure 7B**).

Taken together, these data suggested that HTG and ANP might play a vital role in leading to the changes in structure of intestinal microbiota.

### Antimicrobial Peptides Expression Decreased in HTG-Related ANP

**FIGURE 8:**
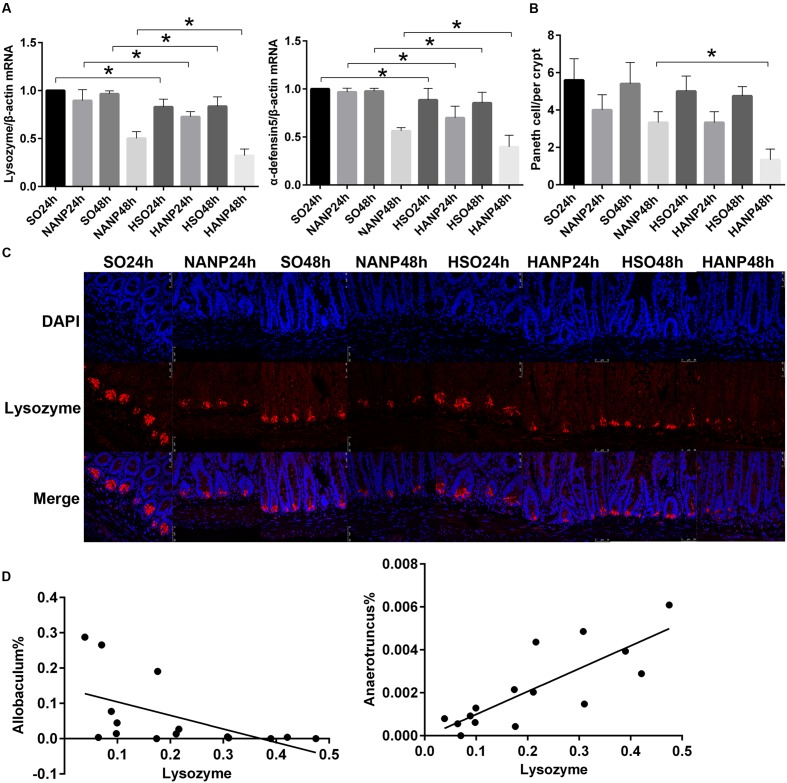
**Antimicrobial peptides expression decreased in HTG-related ANP.** **(A)** Antimicrobial peptides (lysozyme and α-defensin5) mRNA expression by real-time PCR in the distal ileum at 24 and 48 h after ANP induction. **(B)** Quantification of the Paneth cell numbers among the eight groups. **(C)** Lysozyme (red) protein expression produced by Paneth cells in the distal ileum by immunofluorescence. Nuclei were counterstained blue with DAPI (original magnification, ×200). **(D)** The abundance of *Allobaculum* (*r* = –0.661, *p* < 0.05) and *Anaerotruncus* (*r* = 0.793, *p* < 0.05) significantly correlated with lysozyme expression in the NANP48h and HANP48h groups. ^∗^*p* < 0.05. Student’s *t*-test and Spearman test.

To investigate the alterations in Paneth cell AMPs expression level during HANP, real-time PCR and immunofluorescence were used to measure the mRNA and protein expression levels of lysozyme and α-defensin5 in the distal ileum. By real-time PCR, lysozyme and α-defensin5 mRNA expression levels decreased after ANP induction. These two AMPs both decreased significantly in the HSO groups compared with those in the SO groups (*p* < 0.05). In the HANP groups, lysozyme and α-defensin5 mRNA levels significantly decreased compared with those in the NANP groups (*p* < 0.05; **Figure 8A**). The immunofluorescence results manifested decreased Paneth cell lysozyme staining in the distal ileum after ANP induction. The lysozyme staining in the HSO groups decreased compared to that in the SO groups, and it further decreased in the HANP groups compared to that in the NANP groups (**Figure 8C**). The quantification of Paneth cells revealed that the number of Paneth cells decreased after ANP induction. Compared with that in the SO groups, Paneth cells number decreased in the HSO groups, while showing no statistical difference. In the HANP48h group, Paneth cells number significantly decreased compared to that in the NANP48h group (**Figure 8B**). In addition, we found that in the NANP48h and HANP48h groups, the abundance of *Allobaculum* was correlated inversely with lysozyme expression level (*r* = -0.943, *p* < 0.05), while the abundance of *Anaerotruncus* was correlated positively with lysozyme expression by Spearman test (*r* = 0.886, *p* < 0.05; **Figure 8D**).

## Discussion

HTG is now considered as a risk factor which proved to aggravate pancreatic injuries in experimental study (Zeng et al., 2012). Intestinal barrier dysfunction is increasingly acknowledged as a major reason for complications of ANP (Capurso et al., 2012). The excessive release of inflammation cytokines during AP is one of the primary reasons for intestinal barrier injury (Zhang et al., 2007). In our study, HANP rats had more serious pancreatic damage and systemic inflammation than that of the normal lipid rats with ANP, which was similar to the previous study (Zheng et al., 2016). In addition, HANP rats also had higher levels of intestinal proinflammatory cytokine (TNFα, IL-1β, and IL-17A) and more severe injuries in intestinal barrier.

Intestinal microbiota has been acknowledged as a crucial factor in maintaining intestinal barrier (Ivanov and Littman, 2011). HFD could induce shifts in intestinal microbiota structure (Zhang and Yang, 2016). However, researches on changes in diversity of gut microbiota showed different results. Lecomte et al. (2015) reported an increase in microbial diversity in feces from rats fed with HFD for 16 weeks, while Zhang reported that microbial diversity declined significantly after 2 weeks of HFD feeding (Zhang et al., 2012). In our study, HTG models were established by fed with HFD, including lard and cholesterol, for 2 weeks. Our data demonstrated a significant decrease in microbial diversity in the HTG group. The rats’ chronic consumption of HFD containing sweetened condensed milk and saturated animal fat for 16 weeks resulted in the decreased ratio of Firmicutes/Bacteroidetes (Lecomte et al., 2015). In our study, there was no significant alteration in Firmicutes and Bacteroidetes in HTG group. We discovered decreased abundance of *Candidatus_Saccharibacteria* and Tenericutes. Tenericutes was reported to be decreased in C57BL/6 mice with dextran sulfate sodium salt (DSS)-induced colitis (Nagalingam et al., 2011), but the specific function of Tenericutes in influencing intestine remain further analysis.

The alteration in intestinal microbiota structure was also present in SAP. Similar to the previous studies, our results showed shifts in the relative abundance of specific phylotypes during ANP, although the specific changes were different from what those studies had reported. In our study, we found that the microbial diversity in the HANP group decreased significantly compared with that in the NANP group and the structure of intestinal microbiota also had shifts. In consistence with the difference between the normal lipid group and the HTG group, the HANP group also had the decreases in *Candidatus_Saccharibacteria* and Tenericutes in phyla level. In genus level, the HANP group had the decreased abundance of *Alloprevotella*, *Anaerotruncus*, *Christensenellaceae_R-7_group*, and several genera from Ruminococcaceae family and the increased abundance of *Allobaculum* and *Parasutterella. Alloprevotella* is a recently identified genus and was found in isolates from endodontic infections in active dental caries (Schulze-Schweifing et al., 2014). *Allobaculum* was once reported to be increased in mice fed low-fat diet with 3% grapes for 11 weeks, indicating its potential association with improved metabolic health (Baldwin et al., 2016). *Parasutterella* was found to be increased in submucosal tissues of patients with advanced Crohn’s disease (Chiodini et al., 2015). Although the specific role of each intestinal microbiota mentioned above in HANP is unknown, our results confirmed the overall alteration in the structure of intestinal microbiota.

Paneth cells shape and influence structure of intestinal microbiota by secreting a variety of AMPs including α-defensin, lysozyme, secretory phospholipase A2, and RegIIIA (Clevers and Bevins, 2013). The malfunction of Paneth cells has tight relationship with impairment of intestinal barrier, leading to bacterial translocation. In a previous research, Teltschik et al. (2012) investigated the function of Paneth cells in rats with cirrhosis and discovered the reduced AMPs expression such as α-cryptdin5, α-cryptdin7, and lysozyme, which was associated with bacterial translocation. A recent study demonstrated that HFD altered the profile of intestinal microbiota and reduced the expressions of Paneth cell AMPs, which stimulated the intestinal inflammation (Guo et al., 2017). Similar to that study, our results showed that the Paneth cell AMPs (lysozyme and α-defensin5) of the HTG group were at a lower level than that of the normal lipid group. After ANP was induced, these two AMPs further decreased. Compared with the NANP groups, the HANP groups had a significantly decrease in the expression of lysozyme and α-defensin5. Taken together, these findings implied that HTG might aggravate the intestinal barrier dysfunction in HANP by influencing the expression of Paneth cell AMPs.

Furthermore, we analyzed the correlation between microbiota and lysozyme expression level in the NANP and HANP groups. The *Allobaculum* abundance significantly increased in the HANP group and correlated inversely with lysozyme expression. Since *Allobaculum* was considered to play a positive role in intestine in the previous study (Baldwin et al., 2016), we hypothesize that *Allobaculum* may increase in response to the decrease in lysozyme to protect intestine, but further studies are needed to elucidate its specific function during HANP.

## Conclusion

Our findings showed that HANP rats had more severe intestinal barrier injury with increased intestinal permeability and more serious intestinal inflammation. Meanwhile, the shifts in intestinal microbiota and the decreases in Paneth cell AMPs expression were observed in HANP rats, indicating that HTG might aggravate intestine injury by the changes in intestinal microbiota and Paneth cell AMPs expression, resulting in exacerbation of severity during HANP.

## Author Contributions

YZ, XW, and LT designed and conceived the experiments. JC and HZ performed the experiments. JW, LL, and JZ conducted statistic analysis. JC, CH, and YL analyzed data and prepared figures and wrote the paper. YZ and ZC reviewed the paper. All authors carefully read and approved the final version of the manuscript.

## Conflict of Interest Statement

The authors declare that the research was conducted in the absence of any commercial or financial relationships that could be construed as a potential conflict of interest.

## Footnotes

1 http://drive5.com/uparse/

2 http://rdp.cme.msu.edu/

3 www.mothur.org/
