# Supplementary material for: Dysbiosis of Intestinal Microbiota and Decreased Antimicrobial Peptide Level in Paneth Cells during Hypertriglyceridemia-Related Acute Necrotizing Pancreatitis in Rats
Source: Front Microbiol. 2017 May 4;8:776. doi: 10.3389/fmicb.2017.00776 (PMC5415626; doi:10.3389/fmicb.2017.00776)
Supplement: Supplementary file 1 [file Data_Sheet_1.DOCX]

**Dysbiosis of intestinal microbiota and decreased antimicrobial peptide level in Paneth cells during hypertriglyceridemia related acute necrotizing pancreatitis in rats**

Chunlan Huang^1+^, Jing Chen^1+^, Jingjing Wang^2^, Hui Zhou^1^, Yingying Lu^1^, Lihong Lou^3^, Junyuan Zheng^1^, Ling Tian^2^, Xingpeng Wang^1^, Zhongwei Cao^1*^&Yue Zeng^1*^

^1^Department of Gastroenterology, Shanghai General Hospital, Shanghai Jiao Tong University School of Medicine, Shanghai, China. ^2^Shanghai Key Laboratory of Pancreatic Diseases, Shanghai General Hospital, Shanghai Jiao Tong University School of Medicine, Shanghai, China. ^3^International Medical Care Center, Shanghai General Hospital, Shanghai Jiao Tong University School of Medicine, Shanghai, China.

Correspondence and requests for materials should be addressed to Yue Zeng(email: [zengyue1610@163.com](mailto:zengyue1610@163.com)) or Zhongwei Cao(email: caozhongwei@medmail.com.cn)


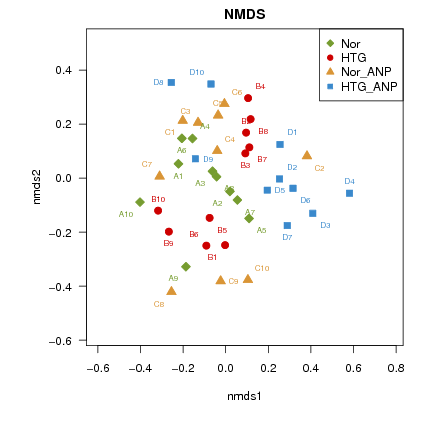


**NMDS based on** Bray-Curtis metrics among the four groups


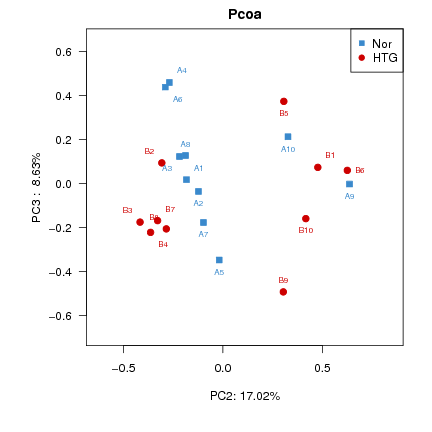


PCoA based on Bray-Curtis metrics between the normal lipid and HTG groups.


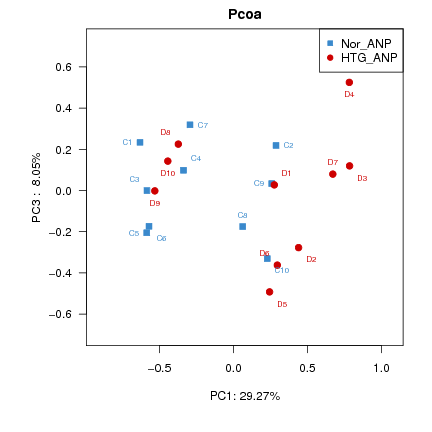


PCoA based on Bray-Curtis metrics between the normal lipid ANP and HTG ANP groups


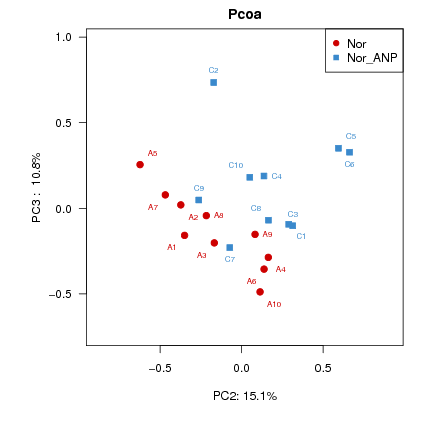


PCoA based on Bray-Curtis metrics between the normal lipid and normal lipid ANP groups


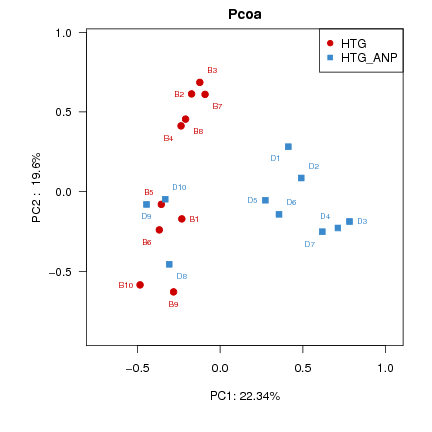


PCoA based on Bray-Curtis metrics between the HTG and HTG ANP groups


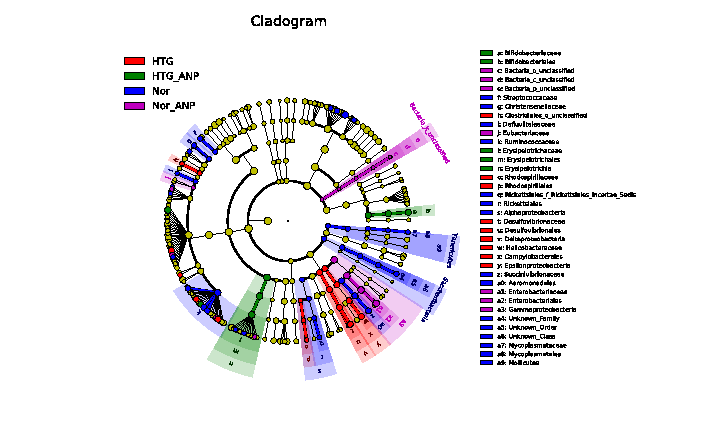

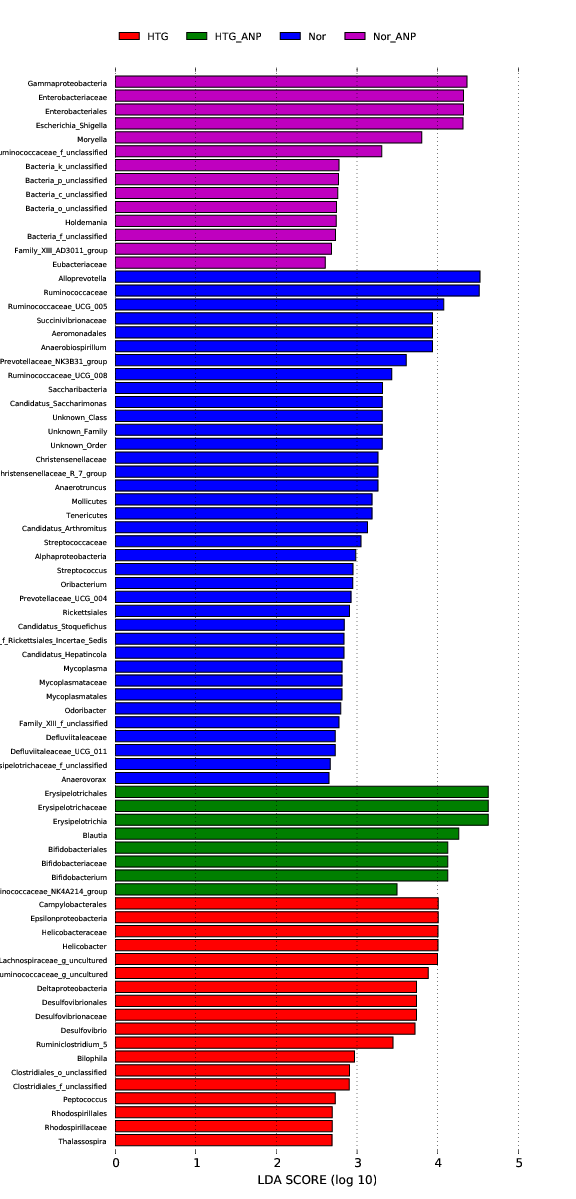


Diﬀerent structure of intestinal microbiota among the four groups by **LEfSe analysis**
